# Organotypic Culture of Neonatal Murine Inner Ear Explants

**DOI:** 10.3389/fncel.2019.00170

**Published:** 2019-05-03

**Authors:** Jacqueline M. Ogier, Rachel A. Burt, Hannah R. Drury, Rebecca Lim, Bryony A. Nayagam

**Affiliations:** ^1^Department of Genetics, The Murdoch Children's Research Institute, Parkville, VIC, Australia; ^2^Department of Paediatrics, The University of Melbourne, Parkville, VIC, Australia; ^3^Department of Genetics, The University of Melbourne, Parkville, VIC, Australia; ^4^School of Biomedical Sciences and Pharmacy, The University of Newcastle, Callaghan, NSW, Australia; ^5^Department of Audiology and Speech Pathology, The University of Melbourne, Parkville, VIC, Australia; ^6^The Bionics Institute, East Melbourne, VIC, Australia

**Keywords:** organ of Corti, peripheral vestibular organs, dissection, cochlea, hair cell culture, mouse, immunohistochemistry, inner ear

## Abstract

The inner ear is a complex organ containing highly specialised cell types and structures that are critical for sensing sound and movement. *In vivo*, the inner ear is difficult to study due to the osseous nature of the otic capsule and its encapsulation within an intricate bony labyrinth. As such, mammalian inner ear explants are an invaluable tool for the study and manipulation of the complex intercellular connections, structures, and cell types within this specialised organ. The greatest strength of this technique is that the complete organ of Corti, or peripheral vestibular organs including hair cells, supporting cells and accompanying neurons, is maintained in its *in situ* form. The greatest weakness of *in vitro* hair cell preparations is the short time frame in which the explanted tissue remains viable. Yet, cochlear explants have proven to be an excellent experimental model for understanding the fundamental aspects of auditory biology, substantiated by their use for over 40 years. In this protocol, we present a modernised inner ear explant technique that employs organotypic cell culture inserts and serum free media. This approach decreases the likelihood of explant damage by eliminating the need for adhesive substances. Serum free media also restricts excessive cellular outgrowth and inter-experimental variability, both of which are side effects of exogenous serum addition to cell cultures. The protocol described can be applied to culture both cochlear and vestibular explants from various mammals. Example outcomes are demonstrated by immunohistochemistry, hair cell quantification, and electrophysiological recordings to validate the versatility and viability of the protocol.

## Introduction

An obstacle faced by inner ear biologists worldwide, is that hair cells from the inner ear cannot be immortalised. In the absence of immortal hair cell lines, the House Ear Institute-Organ of Corti 1 (HEI-OC1) auditory cell line has been used extensively when performing cytotoxic assays, screening protective compounds, elucidating molecular channel and receptor function and protein expression analysis (Kalinec et al., 2016; Park et al., 2016). Whilst serving an important purpose for screening assays, there are significant limitations when using HEI-OC1 for auditory research. Notably, HEI-OC1 cells do not represent a specific auditory phenotype, nor are they able to achieve the complex cell-to-cell interactions present within the highly organised organ of Corti or vestibular sensory epithelium. Moreover, without the ability for specialised mechano-electric transduction, HEI-OC1 drug uptake patterns differ significantly from that of genuine inner ear hair cells. Drug responses vary depending on cell culture conditions and genetic drift has been problematic in the culture of HEI-OC1 (Kalinec et al., 2016). Over time, HEI-OC1 cells have developed drug resistance and have lost some key hair cell characteristics, such as prestin expression which is normally present in cochlear outer hair cells (Cederroth, 2012; Walters et al., 2015; Kalinec et al., 2016). As such, fundamental research in the field of auditory biology relies extensively on the use of mammalian inner ear explant preparations.

*Ex vivo* explant cultures maintain the entire organ of Corti or vestibular epithelium including the delicate inner ear hair cells, supporting cells and neurons in a three-dimensional form, akin to their *in situ* counterparts. As a result, the highly organised, dynamic microenvironment including the specialised cell types that reside within it, remain intact. Preservation of intercellular connections and complex structures provide greater insight into cell-specific stress responses than reduced preparations such as dissociated cell cultures. For example, Wu et al. (2015) were able to extensively characterise the changes in an inner ear explant preparation that were induced by tumour necrosis factor (TNF)-alpha treatment (Wu et al., 2015). In hair cells, changes in stereocilia formation, endoplasmic reticulum components and mitochondrial structures were described. Outer hair cell expulsion from the epithelium was observed as supporting cells attempted to seal the peri-cuticular plate. The chronological order with which cell types (such as pillar cells, Deiters' cells and hair cells) underwent apoptosis, the timing of cell junction degradation, and the presence of factors such as caspase 3 was also shown. This example highlights the incredible versatility and usefulness of the cochlear explant as an *ex vivo* experimental preparation in which variables can be tightly controlled (Wu et al., 2015). Other modern inner ear explant applications include studies of ototoxicity and screening of potentially otoprotective compounds (Lee et al., 2017; Perny et al., 2017), investigating cell stress effects and death pathways (Jiang et al., 2016; Nicholas et al., 2017), exploring novel regenerative strategies (Nayagam et al., 2013; Gunewardene et al., 2016), and elucidating the functions of specific proteins, channels, receptors, and synapses (Flores-Otero et al., 2007; Brugeaud et al., 2014). Much of what we now know regarding the function and structure of the inner ear, and in particular inner ear hair cells, has been established using *in vitro* inner ear explant preparations (Sobkowicz et al., 1975, 2009b; Nordemar, 1983; Russell and Richardson, 1987; Lim et al., 2011).

As a tool for auditory research, inner ear explants have been in use for over 40 years (Sobkowicz et al., 1975, 2009a,b). Consequently, countless protocols exist for the culture of inner ear explants and many iterations have remained in use for a number of years (Doetzlhofer et al., 2009; Mulvaney and Dabdoub, 2014; Haque et al., 2015; Wu et al., 2015; Nicholas et al., 2017). Herein, we present a modernised technique [adapted for *mus musculus* from *Cavia porcellus* protocols (Coleman et al., 2007; Nayagam et al., 2013)]. This protocol utilises organotypic cell culture inserts and serum free media. The protocol is simpler for the researcher and eliminates steps that increase the likelihood of explant damage. We also provide instructions for the dissection of mouse neonatal inner ear organs. The culture method may be applied for the culture of both cochlear and vestibular explants from neonatal mice and rats (Coleman et al., 2007; Nayagam and Minter, 2012; Nayagam et al., 2013). Methods for immunohistochemistry, imaging, hair cell quantification and electrophysiological recordings are provided. Illustrative results are included to demonstrate the versatility and efficacy of this protocol.

### Advantages Afforded by This Protocol

The inner ear is a complex 3D structure, where cell patterning and communication is critical for cell survival. The use of organotypic cell culture inserts protects the explant tissue sample during media changes, prevents drying of the tissue during prolonged culture, provides excellent diffusion of soluble growth factors to the tissue across the substrate and provides uniform structural support. The porous membrane allows cellular outgrowth, whilst limiting the cell layering that can occur on impermeable surfaces. The membrane can be easily cut away to facilitate transfer of the explants for physiological recordings, immunohistochemistry, and/or further analyses.

#### Organotypic Cell Culture Inserts Eliminate the Requirement for Adhesive Substances, Thereby Improving Explant Quality

Cell culture inserts eliminate the need for an adhesive substance, which saves time, reduces the likelihood of damage, and is simpler for the user. Conversely, commonly used adhesives such as collagen, Matrigel or Cell Tak can be particularly unforgiving for the user. For instance, unintentional contact with adhesive when orienting the explant can fix the tissue in an unusable position. Even when placed appropriately, the action of pressing an explant onto an adhesive substance is likely to cause damage and uneven structural support, which is particularly problematic for functional studies. When orienting an explant onto the cell culture insert in media, there is no risk of accidental adhesion and the explant can be easily maneuvered into position without causing damage to delicate cell types.

#### Serum Free Media Improves Experimental Consistency

Animal sera are highly variable and poorly defined mixtures of hormones, vitamins, lipids, transport proteins, binding factors, proliferation factors, growth factors, and other unknown components. Batches from the same supplier are highly variable. Therefore, the addition of serum impairs assay efficacy, compromises experimental consistency and increases the likelihood of pathogenic contamination (Gstraunthaler et al., 2013).

Serum components can also induce undesirable immune responses and encourage inconsistent cellular proliferation (Harrill et al., 2015). For example, in early post-natal cochlear explant preparations the addition of serum encourages proliferation of dividing cell types in the organ of Corti and supporting cell differentiation. As a result, the ultrastructure of the four rows of hair cells is disrupted more quickly than observed in the absence of serum (Nayagam et al., 2013). Additionally, animal sera are expensive and subject to global shortages. In contrast, serum-free media contains consistent nutritional and hormonal formulations, which improves assay sensitivity and supports more homogeneous and reproducible cell assays (Harrill et al., 2015).

### Limitations When Culturing Inner Ear Tissues

#### Inner Ear Hair Cell Survival Is Limited *In vitro*

Optimal inner ear hair cell survival in culture is considered to be < 1 week, however longer culture periods have been reported in both mouse and rat explants (Flores-Otero et al., 2007; Nayagam et al., 2013). A 72 h time-point has been applied in the ototoxic assays described herein. However, survival of hair cells has been described after up to 14 days in culture (Sobkowicz et al., 1975; Nayagam et al., 2013).

#### Neonatal Explants Contain Immature Cell Types

*In vivo*, inner ear hair cells are terminally differentiated by embryonic day 14.5, however a period of quiescence may exist in the early postnatal stages. Prior to the onset of hearing at around postnatal day 12, immature hair cells are capable of spontaneous firing. Auditory ganglia continue to proliferate and embryonic protein expression may persist (Kamiya et al., 2001; Marcotti et al., 2003). Therefore, the developmental age of tissue should be considered when evaluating responses in the inner ear explant preparation *in vitro*. For instance, cultured inner ear hair cells may retain some regenerative capacity and protein expression will vary between explants derived between P1 and P5 (Walters and Zuo, 2013; Cox et al., 2014).

#### Cochlear Explants Contain Resident Macrophages

Macrophage activity within the explant can activate both pro-survival and pro-death signals *in vitro*. Whilst this may complicate experimental outcomes, it is important to consider that similar macrophage activity occurs *in vivo* (reviewed in Francis and Cunningham, 2017).

The addition of serum to culture media can drastically enhance the activity of macrophages *in vitro*, creating less homogenous inflammatory populations and introducing variability to experimental outcomes (refer to advantages of serum-free culture noted above) (Homma and Yamamoto, 1990; Rey-Giraud et al., 2012).

### Foreword

This protocol describes the fundamental procedures for dissecting and culturing early post-natal murine inner ear explants on organotypic cell culture membranes, rather than the glass-based cultures previously described (Parker et al., 2010; Haque et al., 2015). The protocol for culturing explants is relatively simple. However, the initial cochlear dissection is intricate and requires a high degree of skill. A great deal of practice is required to ensure dissected explants are of a quality suitable for use in experimentation. This should be considered when applying for ethics approval or preparing experiments. As an example of the diversity of data that can be acquired using organotypic explant culture, we provide electrophysiological recordings, immunohistochemistry, and quantification of cell survival. Countless alternative techniques can be applied depending on experimental questions. Scanning electron microscopy, RNA sequencing, and complex staining procedures have all been used to elucidate fundamental aspects of cellular biology within the *ex vivo* inner ear explant. However, new techniques will continue to arise, adding value to the inner ear explant as an *ex vivo* model for the study of specialised cell types contained within it.

#### Animal Ethics Approval

All experiments described conform to the Australian Code of Practice for the Care and Use of Animals for Scientific Purposes, 8th edition, 2013. Approval was granted by the animal ethics committees of The University of Newcastle and The Murdoch Childrens Research Institute, in project numbers A766 (MCRI) and A-2013-325 (UoN).

## Materials and Equipment

To complete the dissection described in this protocol a dissecting microscope is required. A laminar flow cabinet or biohazard hood is recommended, but not essential. Recommended dissection tools are curved forceps, scalpels (#10 and #15), pointed forceps (#5 and #55), and a periosteal elevator. However, alternate tools may be used according to user preference. Common lab items are required, such as sterile Petri dishes, 70% ethanol, disposable cleaning wipes (such as kim-wipes), pipettes (0.1–1,000 μl range), and a CO_2_ incubator. Wide bore 1,000 μl pipette tips are useful. A number of organotypic cell culture membranes are available—with varying pore size or membrane material. However, we routinely use Merck Millipore Millicell® organotypic cell culture inserts. These inserts consist of a solid plastic wall and an optically transparent, hydrophilic, polytetrafluoroethylene membrane with a pore size of 0.4 μm (Biopore™). Further equipment and reagents required for inner ear explant culture, staining and mounting are listed below (Table 1).

**Table 1:** Recommended reagent list for the dissection and culture of mammalian inner ear hair cells.

**Reagent**	**Supplier**	**Catalogue number**	**Recommended storage**
MEM	Life Technologies	42360-032	49 ml aliquots at −20°C
DMEM/F12/GlutaMAX	Thermofisher	10565-018	4°C
Non-essential amino acids	Life Technologies	11140-050	500 μl aliquots at 4°C
Pen/Strep	Life Technologies	15140-148	500 μl aliquots at −20°C
Penicillin	Sigma Aldrich	P3032	Aliquots at −20°C
Neurobasal-A medium	Life Technologies	10888-022	49 ml aliquots at −20°C
N2 supplement	Life Technologies	17502-048	500 μl aliquots at −20°C
L-glutamine	Life Technologies	25030-149	500 μl aliquots at −20°C
D-glucose	Life Technologies	15023-021	75 mg aliquots at room temperature
Steriflip disposable vacuum filters	Merck	SE1M179M6	Room temperature
Millicell cell culture insert, 30 mm, hydrophilic PTFE, 0.4 μm	Merck	PICM03050	Room temperature
6 well culture plate	Thermofisher	140675	Room temperature
4% PFA	As preferred		As preferred
Phosphate buffered saline	As preferred		As preferred
Myosin-VII antibody (Rabbit anti-mouse)	Sapphire Bioscience	PTS-25-6790-C050	−20°C
Goat anti-rabbit IgG HandL Alexa Fluor 488	Sapphire Bioscience	AB150085	−20°C
Prolong diamond anti-fade with DAPI	Life Technologies	P36962	Room temperature
Prolong gold anti-fade with DAPI	Life Technologies	P36941	Room temperature
Phalloidin stain	Thermofisher	A12379	−20°C
PTFE printed Microscope slides	Pro Sci Tech	G35068	N/A
Coverslips	Pro Sci Tech	G417	N/A
*In situ* cell death detection kit	Sigma Aldrich	11684795910	−20°C
Triton X-100	Sigma Aldrich	X100-500ml	Room temperature
Goat Serum	Sapphire Bioscience	AB156046	500 μl aliquots at −20°C
10% neutral buffered formalin	Thermo Fisher Scientific	FNNJJ0185B	Room temperature

## Protocol

### General Set-Up

*Note: A laminar flow cabinet minimises potential sources of airborne contamination, thus maximising the number of useful experimental preparations*. *If a laminar flow cabinet is not available, this protocol should be performed in a room without a thoroughfare. Remember to frequently sterilise surfaces and instruments with 70% ethanol. For wiping surfaces, Kim Wipes are recommended, as regular tissues leave small particles behind*.

Thoroughly clean and sterilise the laminar flow (or work bench) before setting-up the dissection microscope. Once in place, sterilise the microscope paying particular attention to parts that will be frequently used, such as focus controls.

Make a small quantity of fresh Minimal Essential Media (MEM) solution and Neurobasal (NB) solution and/or DMEM/F12 GlutaMAX solution, as per Table 2. Filter sterilise each solution and place on wet ice in the laminar flow hood. Explants that were used for electrophysiological recordings of inner ear hair cells were dissected in glycerol-based Ringer's solution (Ye et al., 2006) (Supplementary Table 1). NB solution is used for cochlear explant culture, whereas DMEM/F12 GlutaMAX solution is used for vestibular explant culture. *Note: Ensure glucose powder is completely dissolved before filter sterilising. Solutions can be stored for 2–3 days at 4*°*C*.

**Table 2:** Recommended solutions required for this protocol.

**MEM solution**		**NB solution (for cochlear preparations)**		**DMEM/F12 GlutaMAX solution (for vestibular preparations)**	
**Reagent**	**Quantity**	**Reagent**	**Quantity**	**Reagent**	**Quantity**
Minimum essential media	49 mL	Neurobasal-A media	49 mL	DMEM/F12 Glutamax	15 mL
Non-essential amino acids	500 μL	N2 supplement	500 μL	D-(+)-glucose	42.73 mg
Penicillin/streptomycin	500 μL	L-glutamine	500 μL	Penicillin	200 μl
		D-glucose (Dextrose) powder	75 mg		

*MEM solution is used in the initial dissection steps. NB solution is used for final cochlear explant dissection and culture. Alternatively, glycerol-based Ringer's solution and DMEM/F12 GlutaMAX solution are used for final vestibular explant dissection and culture, respectively. Separate reagent aliquots for each solution can be stored at −20°C to avoid contamination or freeze thawing of the main stock. Once combined, solutions can be stored for 2–3 days at 4°C*.

Within the laminar flow, sterilise pipettes and dissection tools and allow them to dry. Under sterile conditions, open a 6-well culture plate and Millicell packaging. Place the cell culture inserts into the wells and add 1 ml NB solution (or DMEM/F12 GlutaMAX solution) to each well. Add the media under the membrane by holding the pipette tip against the side of the well and slowly releasing the media. Close the plate and incubate for a minimum of 30 min before commencing culture. For hair cell studies: incubate at 37°C, 5% CO_2_; for neuron specific research: incubate at 37°C, 10% CO_2_.

### Collecting Tissue

Euthanise mouse pups (ideally aged between P0-5) by decapitation (or by alternative ethically approved methodology). Collect heads in a sterile Petri dish and place the closed dish in wet ice.

### Dissecting the Temporal Bones

*Note: Videos of the major steps in the dissection of the inner ear from the temporal bone have been published with detailed instructions for dissection of both the cochlea (Parker et al.*, *2010**; Haque et al.*, *2015**) and vestibular organs (Tung et al.*, *2013**). Alternative tools may be used based on user preference and availability*.

Working quickly in the laminar flow hood:

- Use curved forceps to hold the head in place and bisect the skull with a no.10 scalpel.
- Remove the brain using a gentle a scooping motion with a small periosteal elevator.
- Using the scalpel remove the snout, scrape away the skin, and remove unnecessary bone.
- Place the temporal bones into a fresh, Petri dish containing ice-cold MEM.
- Repeat dissection for all remaining heads. Use multiple Petri dishes to maintain clear media for dissections (4–6 temporal bones per Petri dish).

### Isolating the Inner Ear

Use the dissection microscope with low magnification. In a Petri dish containing fresh, ice-cold MEM solution use #5 forceps to remove the inner ear from the temporal bone. Place the dissected organs into ice-cold, NB solution (cochlear) or glycerol-based Ringer's solution (vestibular) and maintain on ice. Repeat for all remaining temporal bones.

*Note: After 3–4 dissections, the media in the Petri dish will become cloudy and obstruct the users view. Replace with cold, fresh MEM solution as required. Likewise, replace the Petri dish if it becomes scratched*.

### Cochlear Dissection

**Figure 1:**
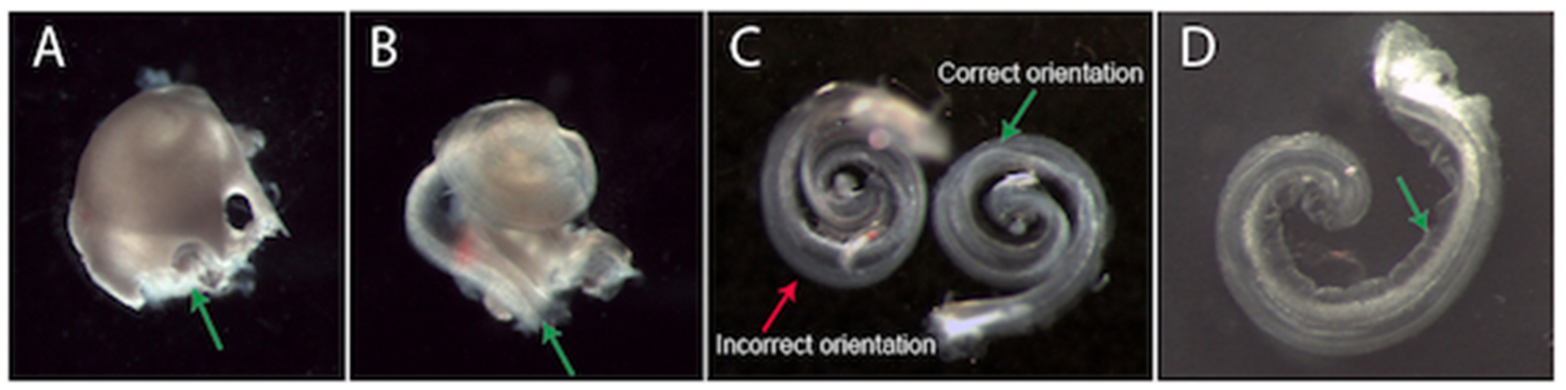
Dissection and plating of a mouse cochlear explant. **(A)** An example cochlea that has been removed from the temporal bone (vestibular apparatus removed). It is easiest to begin removing the bony cochlear wall from the base of the cochlea, starting at either the round or oval window (green arrow, **A**). **(B)** Once the bony cochlear wall is removed, grasp the spiral ligament at the base (green arrow, **B**) and unwind it from the modiolus. *The arrow is pointing at a dark line. This is a dissection “landmark” that will help ascertain a gap between the stria vascularis and neurosensory epithelium. This gap is a good place to start when prising the stria vascularis from the organ of Corti*. **(C)** Two example explants in different positions. The explant on the left is incorrectly oriented with the hair cells touching the surface of the membrane. The explant on the right is in the correct position. When the explant is correctly oriented, Reissner's membrane is clearly visible (green arrow, **C,D**). **(D)** An example explant that has adhered to the Millicell membrane. When excess NB solution is removed, the explant adheres to the Millicell membrane. At this stage, gently moving the explant into a crescent shape (as shown) is recommended. After a few hours incubation, the explant can no longer be moved. *Note: Reissner's membrane (green arrow*, ***D****) has been moved away from the hair cells and has adhered to the Millicell membrane*.

Gently dissect the cochlea from the vestibular apparatus at the promontory (i.e. their junction, Figure 1A) and move the cochlea to a dish containing fresh, ice-cold NB solution.

Increase magnification to improve visualisation of round and oval windows. Use a small picking motion to remove the bone from the cochlea (#55 or jeweler's forceps are ideal). Once the bony cochlear wall is removed, grasp the spiral ligament/stria vascularis at the base- and unwind it from the modiolus (Figure 1B). Carefully secure the basal section of the cochlea with one pair of forceps whilst gently dissecting the basal organ of Corti from the peripheral processes of the auditory neurons in the modiolus.

*Note: If the stria vascularis and organ of Corti are inadvertently unwound from the modiolus together, it is possible to gently separate the tissues in the NB solution*.

### Vestibular Dissection

Using a dissecting microscope, dissect the vestibular organs from the inner ear preparation in an ice-cold slurry of glycerol-based Ringer's solution (Lim et al., 2011). Using forceps to hold the bony cochlea, use a small picking motion to remove bone overlying the anterior and horizontal canals and the vestibule. Remove bone surrounding the anterior and horizontal canals, so that the membranous labyrinth is unobstructed. Once the membranous labyrinth is clearly visible, use fine curved spring scissors to cut and remove the canal membrane close to the neuroepithelium of the anterior and horizontal cristae. Trim and remove the membrane overlying the utricle. Use very sharp spring scissors to cut the overlying membrane, any pulling motion will damage the delicate hair cells. Once the membrane covering the vestibular triad has been removed, continue to remove bone from around the utricle and gently trim the vestibular part of the vestibulocochlear nerve, to free the triad from the bone.

### Transfer the Explants to Millicell Membranes

#### Cochlear Explants

*Note: A video of this step is provided (**Supplementary Video 1**)*.

Using a wide bore and preferably a silicone-coated pipette to collect ~100 μL of NB solution from the dissection dish. Once there is solution inside the pipette, the explant can be drawn into the pipette tip. Expel the explant and media onto the Millicell membrane in one movement. This will result in the explant being suspended within a small liquid bolus with a positive meniscus. Orient the explant into the correct position (Figure 1C). The organ of Corti should be facing upwards with four rows of hair cells visible on the peripheral edge of the explant (as *in situ*).

*Note: The explant is “right side up” if both Reissner's membrane and the tectorial membrane are clearly visible* (Figure 1C). *If the explant is upside down, add more media to orient correctly. The explant is easy to manipulate in a bolus of media*.

Once the explant is in the desired position, use forceps to carefully fold Reissner's membrane away from the hair cells toward the explant modiolus. The tectorial membrane also needs to be gently folded away from the rows of hair cells, towards the explant modiolus, so as to expose their apical stereocilia (start at the apex). Finally, using a smaller pipette, gently aspirate media from the top of the Millicell membrane, starting at the edge of the meniscus*:* the tectorial membrane will fold away from the hair cells and the explant will attach to the Millicell membrane (Figure 1D).

*Note: While a little media remains, the apex and base of the explant can be pushed apart so that the explant resembles a crescent shape rather than a spiral. This makes the apex easier to image- and also limits neurons from growing over the hair cells*. *Be careful to remove any cochlear bone fragments that may have been transferred in the media with the explant*.

*Up to five explants may be cultured on each* Millicell *membrane, however between two to four is ideal*.

#### Vestibular Explants

**Figure 2:**
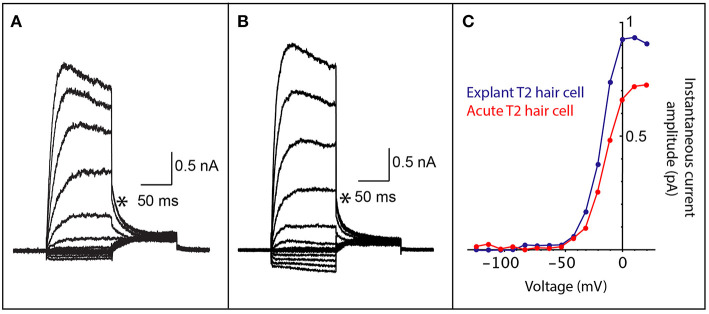
Comparison of whole cell patch clamp recordings from both acutely dissected and explant cultured vestibular hair cells. **(A)** Voltage-activated currents from the crista of a mouse type II vestibular hair cell, prepared as a vestibular triad explant preparation that was cultured for 2 days. **(B)** Voltage-activated currents from the crista of a mouse type II vestibular hair cell from an acute vestibular triad preparation (2 h post dissection). **(C)** An IV plot of instantaneous tail current amplitudes (measured at asterisks in **A,B**) from hair cells in cultured explant preparation (blue trace) and the acute vestibular triad preparation (red trace). The voltage-activated instantaneous tail currents from the explant preparation are comparable to those obtained from an acute preparation and are consistent with recordings from type II vestibular hair cells.

The same technique described above is used for transferring the dissected vestibular triad explant to the Millicell membrane, using a wide bore pipette, filled with DMEM/F12 GlutaMAX culture solution. Ensure the vestibular triad is the right way up. The neuroepithelium that has recently been exposed should face upward, so that the utricle, anterior and horizontal cristae are clearly visible [see Figure 2, Lim et al. (2014)]. The vestibular nerve faces down, resting directly on the membrane. Typically, a triad explant from each ear may be transferred and cultured on a single membrane.

### Incubation

Culture inner ear explants overnight to allow proper adhesion. Treatment can begin immediately if desired by adding to the solution underneath the membrane.

For hair cell studies: incubate at 37°C, 5% CO_2_.

If this protocol is being adapted for inner ear neuron specific research: incubate at 37°C, 10% CO_2_.

### Commence Experimental Treatments

Aspirate the NB or DMEM/F12 GlutaMAX solution and replace with fresh media including treatment or control that has been warmed to 37°C. Replace media daily as desired.

### Electrophysiology

Electrophysiological recordings can be made at any point during the culture time frame but were performed after 2 days *in vitro* in this report. Inner ear explants are transferred to a recording chamber perfused with oxygenated Liebovitz's L15 cell culture media (ThermoFischer Scientific, Australia) with a flow rate of 2–3 bath exchanges/minute. Cells are visualised and targeted for recording using infrared differential interference contrast optics. Borosilicate electrodes (3—5 Mom) are filled with potassium-gluconate internal solution containing (in mM); 42 KGluconate, 98 KCl, 4 HEPES, 0.5 EGTA, 1 Mg_2_ATP, 5 Na_3_GTP. Electrophysiological recordings were done at room temperature using a Multiclamp 700B (Molecular Devices) amplifier, sampled at 20 kHz and filtered at 2–10 kHz and acquired using Axograph software (Molecular Devices). Series resistance was monitored throughout recordings and data was rejected if it increased by more than 20%.

### Fix and Wash Explants for Immunohistochemistry

Aspirate media from each well and replace with 1 mL ice-cold 4% paraformaldehyde (PFA). A drop of fixative on top of each explant is also recommended. Fix at room temperature with *very* gentle rocking for 5–10 min.

*Note: Over-fixation of explants will adversely affect immunocytochemistry. Do not allow explants to dry out at any point during the fixation or staining process*.

Aspirate the fixative and wash each well thrice with 1 mL 1 x Phosphate Buffered Saline (PBS). A drop of PBS should also be added to the top of each explant to rinse away any residual PFA. *Note: At this point slide preparation can commence or the plate can be stored at 4*°*C for up to 3 days, with each well containing PBS -not fixative. For longer-term storage, use a solution of 2% sodium azide in PBS. Note however, that best results for immunohistochemistry are achieved by staining freshly fixed tissues*.

### Immunohistochemistry

#### Myosin-VIIa Inner Ear Hair Cell Stain

Prepare blocking solution (PBS + 2—10% serum relative to secondary antibody+ 0.1% Triton X) and place on roller mixer. While the blocking solution mixes, label Teflon printed slides and add 20 μl 1x PBS to each well on the slide.

*Note: If slides with printed wells are not available, drawing segments on a slide using a PAP pen will suffice. Remember—do not allow explants to dry out*.

Aspirate PBS from each well in the plate and use a #15 scalpel or Vannas micro surgical scissors (WPI) to cut the membrane surrounding each explant. Take care not to fold or tear the membrane whilst doing so. Using forceps, transfer the segments (membrane containing explant) to a well on the printed slide, with the explant facing up. Repeat until all explants have been placed onto slides. Aspirate PBS from each explant on the slide and add 20 μL of blocking solution to each explant. Incubate for 30–60 min at room temperature in a humidified chamber.

*Note: An empty pipette tip container containing water serves as an effective humidified chamber*.

Dilute primary antibody, such as Myosin-VIIa in blocking solution (1:500 for cochlear explants 1:200 for vestibular explants, or per serial dilutions if staining using alternative antibodies). Aspirate blocking solution from explants and pipette 20 μL of the primary antibody solution onto each culture. Incubate for 1 h at room temperature, or overnight at 4°C (cochlear explants) or room temperature (vestibular explants) in a humidified chamber.

Wash explants with 20 μL blocking solution or 0.1 M phosphate buffered saline (PBS) for 2–10 min, aspirate and repeat with fresh blocking solution or PBS three times. To remove any tiny aggregates from the secondary antibody, briefly centrifuge (20 s at ~12,000 g). Dilute secondary antibody 1:200–500 in blocking solution. Pipette 20 μL of secondary antibody solution onto each explant and incubate for 2 h at room temperature (in humidified chamber) or overnight at 4°C. Wash wells with 20 μl blocking solution or PBS for 2–10 min, aspirate and repeat with fresh blocking solution or PBS three times.

*Note: At this point there are several options: keep explants in the final rinse of blocking solution and image as a wet specimen; perform additional staining such as the in situ TUNEL stain (step 13) or apply further antibodies; or, mount under coverslips (step 14)*.

#### Stereocilia Staining Using Phalloidin

Prepare a detergent solution of PBS + 0.1% Triton X and mix on a roller mixer.

While the solution mixes, prepare the explants for slide-based staining as described above. Aspirate PBS from each explant on the slide and add 20 μL of detergent solution to each explant. After 5 min, aspirate the solution and replace with 20 μL detergent solution containing a fluorescently labelled phalloidin (Invitrogen) diluted 1:80 as per manufacturer's instructions). Gently add the detergent/phalloidin mixture in a drop wise manner on top of the explant. Incubate for 10 min and then wash each well thrice for 10 min with 20 μL PBS. *Note: At this point explants can be imaged as a wet specimen or mounted onto glass slides (step 14). Incubating phalloidin on an explant for longer than 15 min will result in extensive over staining of all cell types, preventing clear visualisation of structures*.

### Tunel Stain

Mix the *in situ* cell death detection kit solutions as per manufacturer instructions (Sigma Aldrich). Add 10 μL of the solution to each explant and incubate at 37°C for 1 h (in humidified chamber). Wash wells with 20 μL blocking solution for 2 min, aspirate and repeat with fresh blocking solution three times.

### Mount Explants Under Coverslips and Seal Slides

Aspirate blocking solution and add a *small* drop of Prolong Gold anti-fade mounting medium (with or without DAPI) to each well. Starting at a 45° angle, gently place coverslip on slide. Do not press down or attempt to lift the coverslip once placed. Seal edges with clear nail varnish and allow to dry overnight. Fluorescence will last for a number of months, with slides stored in a lightproof slide box. However, explants should be imaged as soon as possible.

*Note: When higher resolution imaging is being performed (such as stereocilia imaging), Prolong Diamond anti-fade mountant is recommended as it has a refractive index similar to immersion oil. Ensure the selected coverslip thickness is appropriate for the objective being used (most objectives are designed to be used with a #1.5 coverslip—approx. 0.17 mm)*.

## Results

### Physiological Recordings From Inner Ear Hair Cells in Explant Culture

To demonstrate that cell culture with Millicell inserts maintains viable inner ear hair cells, we performed electrophysiological recordings on hair cells from the vestibular apparatus. Electrophysiological recordings are essential for determining whether cells are functionally viable following culture as explant preparations. Whole-cell patch clamp recording gives an indication of the presence and function of voltage-gated ion channels, receptor-mediated currents, intrinsic membrane properties and discharge properties (if recording from neurons), depending on the configuration used. We maintained mouse vestibular explants on Millicell membranes for 2 days in culture and then recorded voltage-activated currents (Figure 2A). These cell responses, which are due to K^+^ channel activation, can only be recorded from cells that are viable. The profile of voltage-activated responses is consistent with recordings from type II vestibular hair cells in an acute semi-intact preparation (Figures 2B,C). Furthermore, the amplitude and kinetics of voltage-activated currents in mouse explants are comparable to those previously studied. All hair cell recordings in explant or acute preparation were from the anterior or horizontal cristae ampullares. These data show the viability of the explant preparation described for electrophysiological recordings. Recordings are not possible from cells that are unhealthy or dead since the cell membrane degrades preventing recording in the whole-cell patch configuration.

### Immunostaining and Cell Viability Assays

Inner ear explants can be labelled using immunofluorescence techniques tailored to highlight particular proteins, structures or regions of interest within the explant- whilst attached to the Millicell membrane.

#### Phalloidin Staining

**Figure 3:**
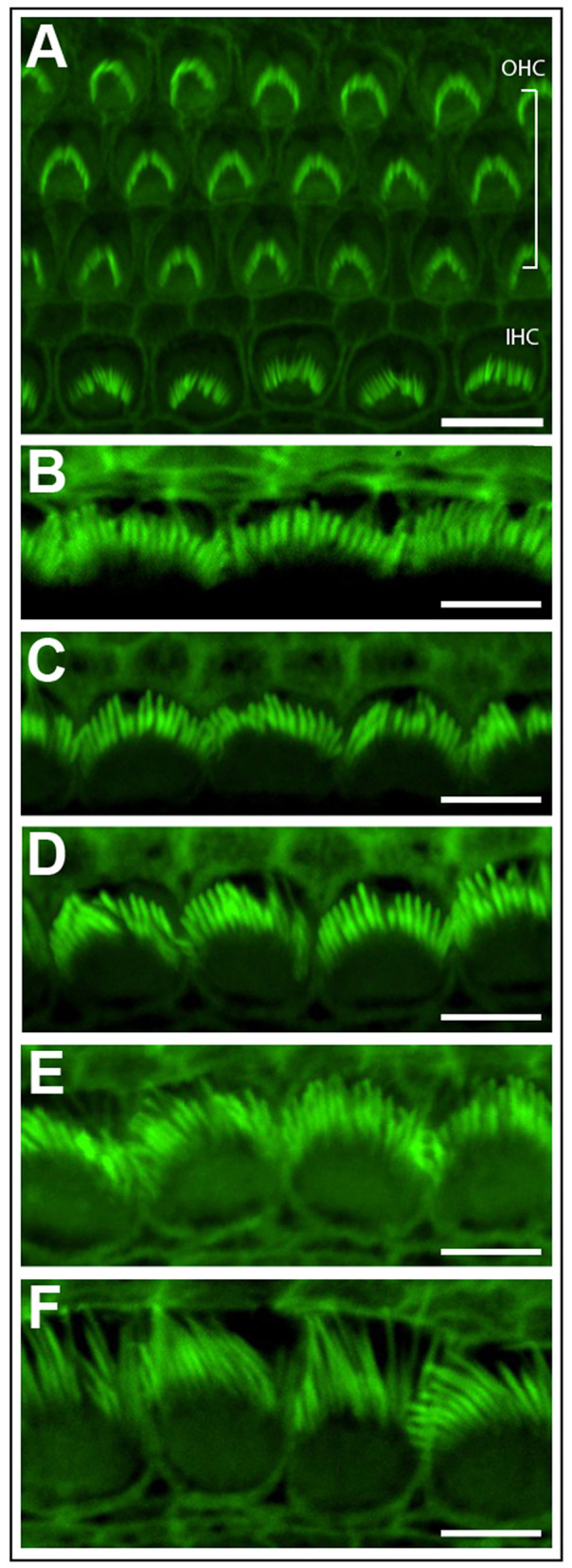
Phalloidin stained cochlear hair cells. **(A)** One row of inner hair cells (IHC) and three rows of outer hair cells (OHC) illustrating normal polarity and patterning of cochlear stereocilia bundles. Scale bar = 10 μm. **(B–F)** The length of inner hair cell stereocilia (green) increases from the basal to apical regions of the cochlea. Conversely, stereocilia width decreases from the cochlear base to apex. Images were progressively collected from the basal region of the explant **(B)** through to the mid-section **(D)** through to the apex **(F)** to demonstrate the graded change in stereocilia length and width. Scale bar = 5 μm. Tissues mounted using Prolong Diamond anti-fade reagent and coverslip #1.5.

Phalloidin staining can highlight the stereocilia located on the apical surface of inner ear hair cells (Figure 3). This technique is a rapid and cost effective way to observe stereocilia polarity and patterning (Figure 3A), before committing to more expensive techniques such as scanning electron microscopy. Subtle changes in stereocilia morphology, such as the increase in their length from the base to apex in the cochlea can be observed. In the cochlear basal turn, the stereocilia are very short (Figure 3B), however stereocilia length increases towards the cochlear apex (Garfinkle and Saunders, 1983) (Figure 3F). Conversely, stereocilia width gradually decreases from the basal to apical regions of the cochlea (Sekerková et al., 2011).

#### TUNEL Staining

**Figure 4:**
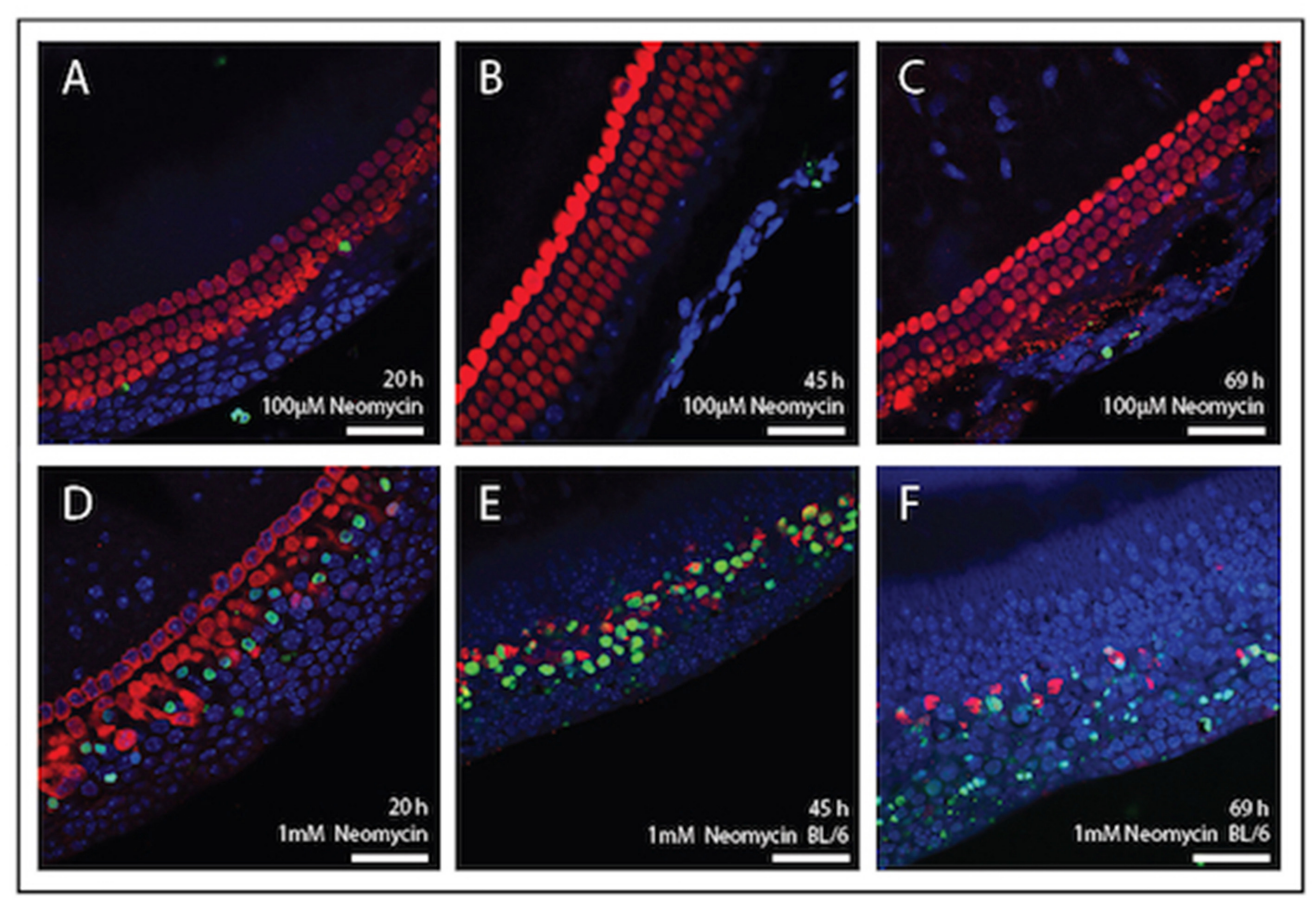
Immunolabelling of inner ear hair cells from mouse cochlear explant preparations after, 20, 45, and 69 h in culture. Cochlear explants were treated with the ototoxic drug, neomycin. Changes in cellular morphology and DNA damage indicative of apoptosis were detected using the TUNEL assay (green). DAPI (blue) was used to stain all cell nuclei and Myosin-VII (red) specifically stained surviving hair cells. **(A–C)** Explants treated with 100 μM neomycin (20, 45, and 69 h). **(D–F)** Explants were treated with 1 mM neomycin (20, 45, and 69 h). Scale bar = 40 μm. Tissues mounted using Prolong Gold anti-fade reagent and coverslip #1.

Figure 4 shows mouse cochlear explants that have been treated with the ototoxic drug neomycin. In this example immunohistochemistry was used to visualise the cochlear hair cells and a fluorescent TUNEL stain was used to highlight cells undergoing apoptosis. Structural changes associated with cell death, such as cell condensation are visible and the TUNEL stain clearly indicates the presence of degraded DNA in apoptotic cells. Such cell death, or cell survival can then be quantified according to treatment and timeframe and is outlined below.

### Quantification

**Figure 5:**
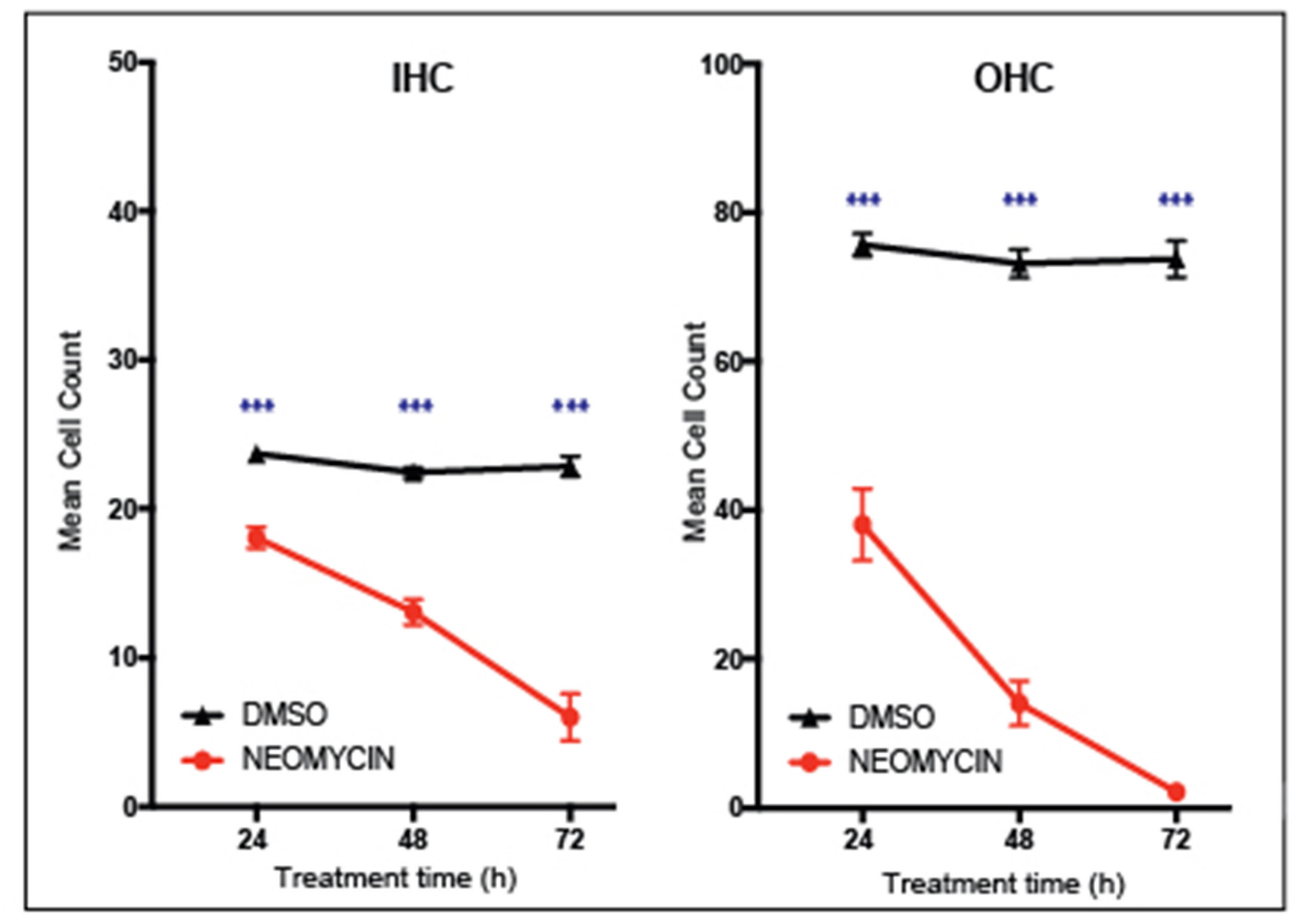
Inner ear hair cell survival over a 72-h time course. *In vitro* hair cell counts were performed 24, 48, or 72 h after treatment with either 1 mM neomycin, or Dimethyl sulfoxide equivalent (20 μl DMSO in 992 μl media). Cell counts are based on the average number of Myosin VIIa-positive hair cells counted in two 180 × 90 um sections of the cochlear explant mid-turn. Mean with standard error of the mean (SEM) shown, *n* = 6–9 explants per treatment, ^***^p < 0.001.

Performing cell counts is an important step for quantifying cell survival under a range of experimental conditions. Cell counts can be a particularly powerful way to show the effects of a compound on hair cell viability. For example, Figure 5 shows that the aminoglycoside antibiotic neomycin is ototoxic, destroying cochlear hair cells. After 3 days treatment with 1 mM neomycin, 98 % of outer hair cells were destroyed whereas inner hair cells were less affected with 67% destroyed after 3 days of neomycin treatment. Using this technique, it is then possible to quantify positive effects of putative otoprotective compounds. Any section of the explant can be counted, however it is important to note the hair cells in the apical region are highly resistant to ototoxicity, due to reduced drug uptake. Conversely, cells in the basal region are more susceptible to cell death and are also more likely to be damaged during dissection.

## Summary

We have described the fundamental procedures for dissecting, plating, and culturing early post-natal murine inner ear explants using Millicell culture inserts and serum- free conditions. The use of serum free media for cochlear and vestibular explant culture is becoming more common (Kopke et al., 1997; Cunningham et al., 2002; Amarjargal et al., 2009; Bas et al., 2014; Glueckert et al., 2015; Wu et al., 2018). And, published methods similar to this protocol further validate the efficacy of organotypic cell culture inserts when culturing cochlear or vestibular tissues (Praetorius et al., 2010; Baker and Staecker, 2012; Dammeyer et al., 2014; Defourny et al., 2015; Ma et al., 2017). In our hands, this protocol has been used to culture both cochlear and vestibular explants derived from rats and mice (Coleman et al., 2007; Nayagam et al., 2013; Gunewardene et al., 2016). We have provided examples of cell viability and function using patch-clamp electrophysiology, immunohistochemistry, and quantification. Alternative techniques can be readily applied to the described explants depending upon specific experimental questions. It is important to remember that, whilst the protocol is simple to follow, the dissections described require patience and practice. The use of Millicell culture inserts described in this protocol eliminates the need for adhesive substances whilst still anchoring the explant. As a result, important experimental tissues are not inadvertently damaged or lost during initial adhesion or subsequent media changes. The explant can be easily maneuvered into position with minimal direct contact and the Millicell membrane provides uniform structural support during media changes and culture. Additionally, the use of serum free media restricts excessive cellular outgrowth and inter-experimental variability.

## Ethics Statement

All experiments described conform to the Australian Code of Practice for the Care and Use of Animals for Scientific Purposes, 8th edition, 2013. Approval was granted by the animal ethics committees of The University of Newcastle and The Murdoch Childrens Research Institute, in project numbers A766 (MCRI) and A-2013-325 (UoN).

## Author Contributions

JO, RB, RL, and BN conceived the experiments. JO, BN, and RL wrote the manuscript. JO prepared all figures for publication. HD and RL performed the vestibular explant experiments and produced the diagrams presented in Figure 2. JO conducted all cochlear explant experiments producing the data and diagrams presented in Figures 3–5. Cochlear dissections for Figure 1 images and Supplementary Video 1 were performed by BN and edited for publication by JO. All authors reviewed the manuscript.

### Conflict of Interest Statement

The authors declare that the research was conducted in the absence of any commercial or financial relationships that could be construed as a potential conflict of interest.
