# Corrigendum: Organotypic culture of neonatal murine inner ear explants

**DOI:** 10.3389/fncel.2024.1512599

**Published:** 2024-11-14

**Authors:** Jacqueline M. Ogier, Rachel A. Burt, Hannah R. Drury, Rebecca Lim, Bryony A. Nayagam

**Affiliations:** ^1^Department of Genetics, The Murdoch Children's Research Institute, Parkville, VIC, Australia; ^2^Department of Paediatrics, The University of Melbourne, Parkville, VIC, Australia; ^3^Department of Genetics, The University of Melbourne, Parkville, VIC, Australia; ^4^School of Biomedical Sciences and Pharmacy, The University of Newcastle, Callaghan, NSW, Australia; ^5^Department of Audiology and Speech Pathology, The University of Melbourne, Parkville, VIC, Australia; ^6^The Bionics Institute, East Melbourne, VIC, Australia

**Keywords:** organ of Corti, peripheral vestibular organs, dissection, cochlea, hair cell culture, mouse, immunohistochemistry, inner ear

In the published article, there was an error in Table 2. The amount of D-glucose to include in NB solution was incorrectly listed as “75 ug.” The correct amount is “75 mg.” The corrected Table 2 and its caption appear below.

**Table 2:** Recommended solutions required for this protocol.

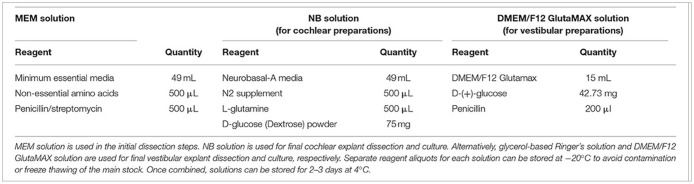

The authors apologize for this error and state that this does not change the scientific conclusions of the article in any way. The original article has been updated.

