# Supplementary material for: Organotypic Culture of Neonatal Murine Inner Ear Explants
Source: Front Cell Neurosci. 2019 May 3;13:170. doi: 10.3389/fncel.2019.00170 (PMC6509234; doi:10.3389/fncel.2019.00170)
Supplement: Supplementary file 1 [file Table_1.DOCX]

**Supplementary Table 1 :** Glycerol-based Ringers solution used during the dissection of vestibular explants, as described by Ye et al*.* (Ye et al. 2006)*.*

| CHEMICAL | MOLARITY (mM) |
| --- | --- |
| NaHCO_3_ | 26 |
| Glucose | 11 |
| Glycerol | 250 |
| KCl (1M stock) | 2.5 |
| NaH_2_PO_4_ (1M stock) | 1.2 |
| MgCl_2_ (1M stock) | 1.2 |
| CaCl_2_ (1M stock) | 2.4 |
